# A cross-sectional study on temporal distribution characteristics of outpatient visits in a tertiary TCM hospital: Implications for nursing staff allocation

**DOI:** 10.1097/MD.0000000000050301

**Published:** 2026-08-14

**Authors:** Qin Wang, Chunchun Chen, Huayu Fang, Jing Meng, Yan Cao, Xiaofang Wang, Zhigang Lin

**Affiliations:** a Outpatient Department, Affiliated Rehabilitation Hospital of Fujian University of Traditional Chinese Medicine, Fuzhou, Fujian Province, China; b Nursing Department, Affiliated Rehabilitation Hospital of Fujian University of Traditional Chinese Medicine, Fuzhou, Fujian Province, China; c Department of Hospital Administration, Affiliated Rehabilitation Hospital of Fujian University of Traditional Chinese Medicine, Fuzhou, Fujian Province, China.

**Keywords:** circular distribution method, concentration degree method, log-linear regression, nursing resource allocation, outpatient visit, temporal distribution, Traditional Chinese Medicine (TCM)

## Abstract

To identify temporal distribution patterns and peak periods of outpatient visits in a tertiary grade A Traditional Chinese Medicine (TCM) hospital and to provide a reference for the formulation of outpatient nursing staffing plans, we conducted a retrospective analysis of daily outpatient data collected from January 2020 to December 2024. Visits from walk-in clinics and physical examination departments were excluded to eliminate confounding factors related to COVID-19 nucleic acid testing and ensure the dataset reflected routine TCM clinical needs. Log-linear regression, the concentration degree method (*M* value), and the circular distribution method were adopted for data analysis, and all statistical analyses were performed using R software (version 4.4.1). Annual outpatient volume showed a statistically significant steady upward trend, with an annual growth rate of 9.92% (β = 0.0947, 95% CI: 0.0814–0.1080, *P* < .001). The *M* values ranged from 0.179 to 0.330 across the 5 years, with an overall *M* value of 0.218. Circular distribution analysis identified October 15 as the overall peak day, with the peak period falling on October 13 to 18; annual peaks were mainly concentrated between mid-September and early November. Rayleigh tests revealed statistically significant temporal aggregation (all *P* < .001). The corresponding *r* values ranged from 0.034 to 0.184, indicating weak practical aggregation intensity of outpatient visits. TCM outpatient visits presented steady growth and autumn-centered temporal distribution characteristics. We put forward potential ideas for outpatient nursing staffing planning, which can serve as a reference for clinical practice and facilitate the integration of the TCM concept of “Zhi Wei Bing (preventive treatment of disease)” into daily work.

## 1. Introduction

Outpatient nursing in tertiary grade A Traditional Chinese Medicine (TCM) hospitals often confronts seasonal service bottlenecks, which directly correlates with diminished nursing satisfaction and imbalances between nursing resource supply and demand.^[1]^ This challenge stems inherently from the temporal distribution of outpatient visits. Guided by TCM’s core theory of “Tian Ren Xiang Ying (correspondence between heaven and humans [a core TCM concept emphasizing the inherent unity and dynamic interaction between the human body and nature, including seasonal rhythms, climatic changes, and natural laws]),” human health is dynamically linked to natural seasonal fluctuations, resulting in distinct patterns of outpatient demand.^[2]^ Temporal demand modeling has long been a core research direction in health services management. Relevant studies have confirmed its value for medical demand forecasting, while its practical effects on optimizing service efficiency and balancing supply and demand vary across different healthcare settings.^[3]^ Classic studies have confirmed that scientific temporal distribution analysis of medical service demand is the premise of rational resource scheduling, which provides a methodological framework applicable to medical institutions with different scales and service scopes.^[4]^ Analyzing these temporal regularities and identifying peak visit periods is therefore critical to addressing nursing supply-demand mismatches, enhancing service efficiency, and advancing data-driven nursing resource management.^[5,6]^

Notably, the TCM theory of “Qiu Zhuo Zao Qi (dryness dominates autumn [a core TCM seasonal health concept indicating dryness as the predominant pathogenic factor in autumn that impairs human health])” may partly explain the rise in outpatient visits during autumn. Dryness in this season is likely to induce respiratory diseases, xerosis syndrome, dermatological disorders, and mood disorders.^[7]^ Meanwhile, growing public attention to TCM health preservation ideas, including “Qiu Dong Yang Yin (nourishing yin in autumn and winter [a classic TCM health preservation concept advocating yin-nourishing therapies in autumn and winter to adapt to seasonal dryness and cold])” could also contribute to increased demand for specialized TCM services such as herbal paste and moisturizing treatments.^[8,9]^

Most existing research on outpatient temporal distribution has centered on Western medical hospitals in China,^[3-6]^ leaving a critical gap in data on TCM-specific visit patterns. Existing studies on Western medicine hospitals in China have mostly adopted the SARIMA model for outpatient volume prediction, which has significant advantages in short-term time series forecasting for outpatient flow with obvious seasonal and periodic characteristics.^[10,11]^ In contrast, log-linear regression is suitable for quantifying the long-term linear growth trend of medical service volume, which matches the research purpose of this study.^[12-15]^ Furthermore, combining the concentration degree method,^[16,17]^ and circular distribution method,^[17]^ with log-linear regression can realize the comprehensive analysis of TCM outpatient volume from the perspectives of long-term trend, seasonality, and temporal aggregation, and make up for the deficiency of single method analysis. Traditional seasonal decomposition primarily describes time periodic fluctuations and cannot separately assess long-term growth trends or pinpoint the exact daily clustering of visits. By comparison, our integrated analytical framework can independently quantify the annual long-term trend, monthly seasonal concentration, and specific daily peak period, which better fits the multidimensional research objectives of this study. To fill this gap, we performed a 5 year retrospective analysis utilizing log-linear regression, the concentration degree method, and the circular distribution method to systematically evaluate long-term trends, seasonality, and peak periods of outpatient visits in a TCM hospital. Our findings seek to deliver evidence-based insights for optimizing outpatient nursing resource allocation in TCM settings, facilitating a transition from “passive response” to “proactive management” in clinical nursing practice.

## 2. Materials and methods

### 2.1. Data source

Data were extracted from the information management system of a tertiary grade A Traditional Chinese Medicine (TCM) hospital, encompassing aggregated department-specific daily outpatient volume counts from all clinical departments at both the main and branch campuses between January 2020 and December 2024. The total number of outpatient visits in the original dataset (including walk-in clinics and physical examination departments) during the study period was 12,29,654 person-times; 393,320 person-times were excluded after screening, and the final valid data of 8,36,334 person-times were included in the subsequent analysis. The inclusion rate was 68.0% (8,36,334/12,29,654). The excluded data mainly included a large number of COVID-19 nucleic acid testing visits in walk-in clinics and physical examination departments during the pandemic period, and the exclusion was to eliminate the confounding interference of pandemic-related testing and ensure the dataset reflected the routine TCM clinical outpatient demand. The dataset contained only numerical summary data (e.g., daily number of visits in the TCM Internal Medicine Department, daily number of visits in the Acupuncture and Moxibustion Department) without any individual patient identifiers (e.g., name, medical record number, contact information, address). Through the hospital’s department management records, we confirmed that there were no new or discontinued clinical departments in the 5 years, and the service scope and business content of each included department remained stable without significant changes, which ensured the consistency of the research objects and the reliability of the trend analysis results. During the study period, statutory public holidays followed a regular annual cycle, and their long-term stable impact would not disturb the analysis of seasonal outpatient distribution. There were no updates to the outpatient booking system and no adjustments to staffing policies throughout 2020 to 2024. Considering dramatic fluctuations in walk-in clinics and physical examination departments due to COVID-19 nucleic acid testing services before and after the pandemic, we have excluded relevant visits to rule out interference from altered medical-seeking behaviors during the pandemic recovery phase and ensure the dataset represents regular TCM outpatient needs. Ultimately, 60 months of monthly aggregated data were utilized for concentration degree analysis, and 1826 days of daily aggregated data were used for circular distribution analysis. All data were processed in compliance with the Declaration of Helsinki and the hospital’s data governance policies, as no individual patient privacy was involved.

#### 2.1.1. Ethical statement

This study was implemented in strict accordance with the Measures for the Ethical Review of Biomedical Research Involving Human Subjects (National Health Commission of China, 2016) and the relevant ethical review regulations of the Affiliated Rehabilitation Hospital of Fujian University of Traditional Chinese Medicine. Since this study only utilized de-identified aggregated data on outpatient visits, did not include any identifiable personal information, and involved no intervention with human subjects, ethical approval and informed consent were waived by the hospital’s ethical review committee. All data collection and analysis procedures complied with national and local regulations.

### 2.2. Study design and statistical methods

#### 2.2.1. Descriptive statistics

Annual and monthly outpatient volumes (after exclusion) were summarized to characterize basic distribution patterns.

#### 2.2.2. Log-linear regression analysis

Log-linear regression was used as the primary method to evaluate the long-term linear trend of annual outpatient volume. The log-transformed annual outpatient volume was used as the dependent variable, and the calendar year was set as the independent variable. The annual growth rate was derived from the regression coefficient, accompanied by a 95% confidence interval (CI) and *P* value.^[12-15]^

#### 2.2.3. Concentration degree method (M value)

The concentration degree (*M* value) was used to evaluate the seasonality of outpatient visits. The *M* value is derived from the vector synthesis of monthly visit proportions (*r_x_*), calculated using the following formulas:^[17]^


$$\begin{array}{cc} & \overset{2}{\underset{X}{R}}=\left(r_{2}+r_{6}-r_{8}-r_{12}\right)/2\\ & +\sqrt{3}\left(r_{3}+r_{5}-r_{9}-r_{11}\right)/2+(r_{4}-r_{10}) \end{array}$$

$$\begin{array}{c} \begin{array}{cc} & R_{y}^{2}=\left(r_{3}-r_{5}-r_{9}+r_{11}\right)/2\\ & +\sqrt{3}\left(r_{2}-r_{6}-r_{8}-r_{12}\right)/2+(r_{1}-r_{7}) \end{array} \end{array}$$

$$\begin{array}{c} M=\sqrt{R_{X}^{2}+R_{y}^{2}} \end{array}$$

Where *r_x_* represents the proportion of outpatient visits in month × relative to total annual visits. The *M* value is a quantitative descriptive indicator to measure the intensity of seasonal aggregation of outpatient visits, and the smaller the value, the weaker the seasonal aggregation of outpatient visits.^[17]^ This study only uses the *M* value for cross-year comparative description of the distribution characteristics of TCM outpatient volume to ensure the scientificity of index application.

#### 2.2.4. Circular distribution method

Daily dates were transformed into annual day-of-year values (1–365) and corresponding angles (0–360°) for circular distribution analysis. For the 2024 leap year, we unified the day-of-year transformation rule: February 29 was assigned a day-of-year value of 60, and the subsequent dates followed the same assignment standard as non-leap years; the day-of-year values of all study years were standardized to 1 to 365 to ensure cross-year consistency. We verified the unimodal distribution assumption of the Rayleigh test for each year’s outpatient volume distribution using the kernel density estimation method. Silverman rule-of-thumb was adopted for bandwidth selection in kernel density estimation to enhance the reproducibility of analyses. The results confirmed that all annual distributions were unimodal and met the test assumption. In addition, considering the low *r* values of temporal aggregation, we supplemented the Watson uniformity test as a conservative verification for all annual and total data. Daily outpatient counts are inherently temporally autocorrelated and do not represent independent observations. Such data dependence may interfere with the interpretation of statistical significance in the Rayleigh test, and this limitation is discussed accordingly. The mean angle (corresponding to the peak visit day), angular standard deviation (with the peak period defined as the mean angle ± angular standard deviation), and Rayleigh test (*r*, *Z*, *P* values) as well as Watson uniformity test (*W*, *P* values) were calculated to evaluate the statistical significance of temporal aggregation (detailed formulas are provided in references).^[17]^ Rose charts were used for data visualization.

#### 2.2.5. Statistical analysis

All statistical analyses were performed using R software (version 4.4.1; The R Foundation for Statistical Computing, Vienna, Austria). A log-linear regression model was applied to the time-series data. The regression coefficient, 95% CI, and *P* value were estimated to quantify the linear growth trend and annual growth rate. Combined plots were constructed using the “ggplot2” and “gridExtra” R packages, while rose charts were created using the “ggplot2” and “circular” packages. A 2-tailed *P* value < .05 was considered statistically significant.

## 3. Results

### 3.1. Basic trends of outpatient volume

**Figure 1.**
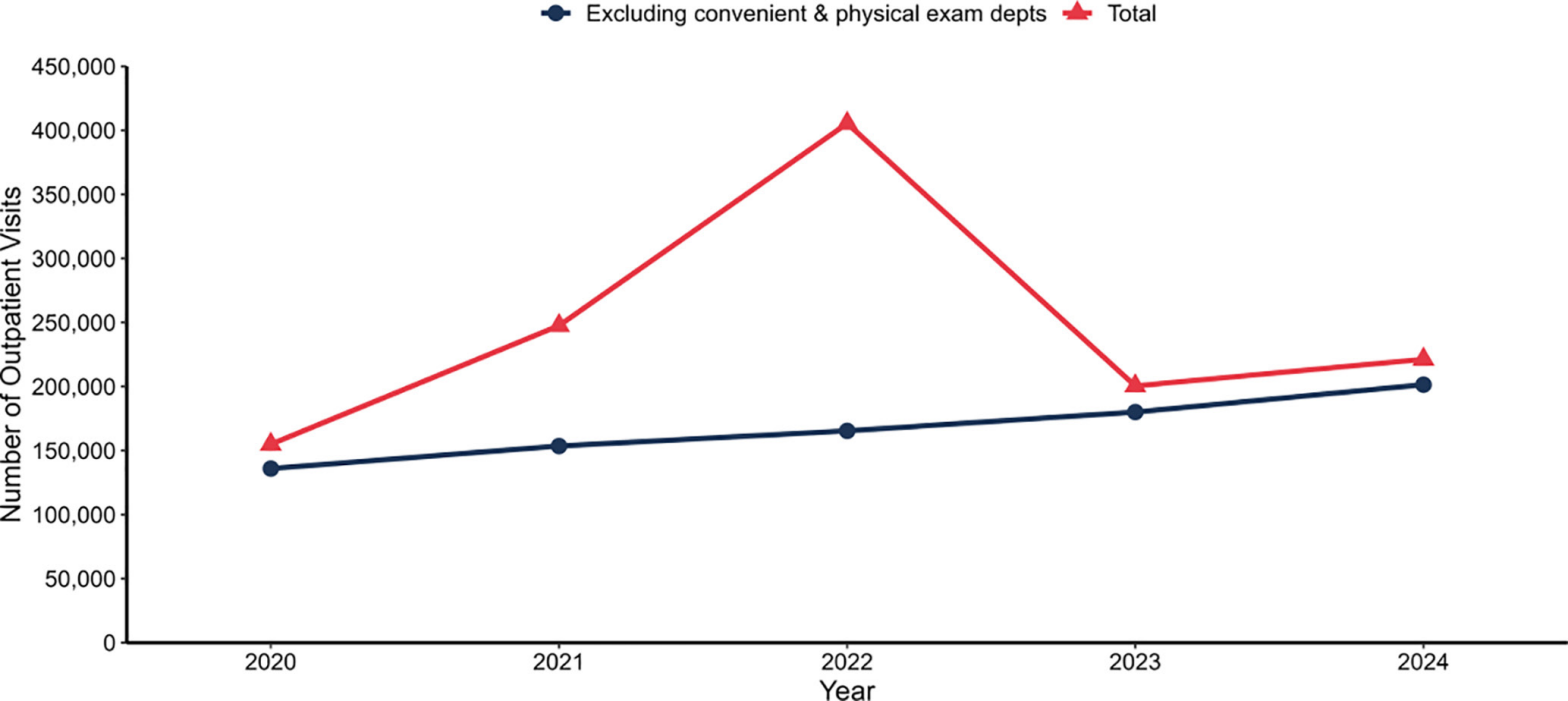
Annual outpatient volume in a tertiary TCM hospital (2020–2024). Annual outpatient volume of the hospital (excluding walk-in clinics and physical examination departments) from 2020 to 2024, showing a continuous upward trend. TCM = Traditional Chinese Medicine.

The annual outpatient volume exhibited a stable increasing trend between 2020 and 2024 after excluding data from convenient walk-in clinics and physical examination departments (Fig. 1). The annual outpatient volumes recorded were 1,35,952, 1,53,539, 1,65,376, 1,80,037, and 2,02,430 from 2020 to 2024, respectively, which is consistent with the year-by-year upward trend illustrated in Figure 1.

**Figure 2.**
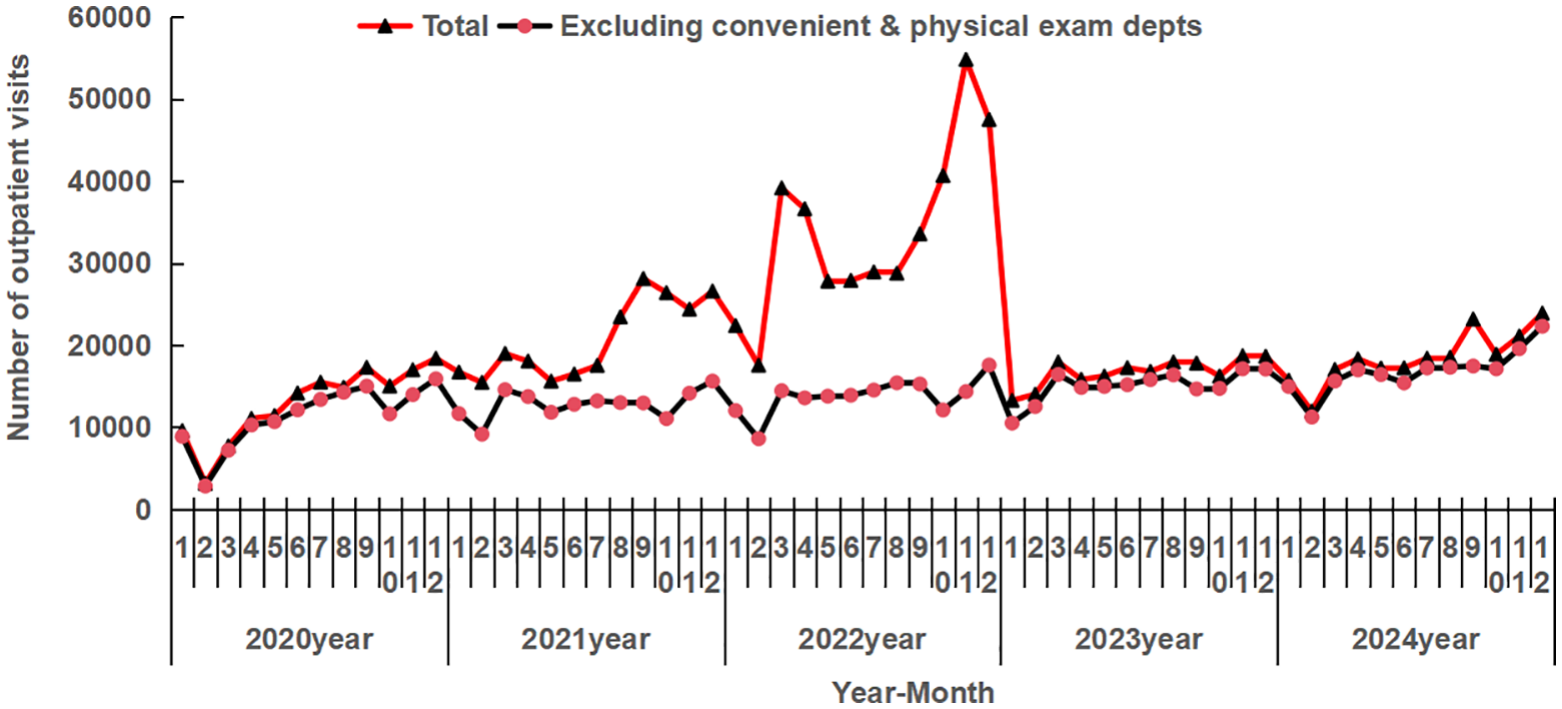
Monthly outpatient volume in a tertiary TCM hospital by year and month (2020–2024). Monthly fluctuations of outpatient volume across 5 years, with consistent peaks in autumn (September–November). TCM = Traditional Chinese Medicine.

Monthly outpatient volume presented significant seasonal fluctuation characteristics throughout the study period (Fig. 2). Obvious inter-annual and intra-annual variations were identified in monthly outpatient visits, with the annual volume peak consistently occurring in autumn (September–November) in all observed years (Fig. 2).

### 3.2. Log-linear regression analysis

**Figure 3.**
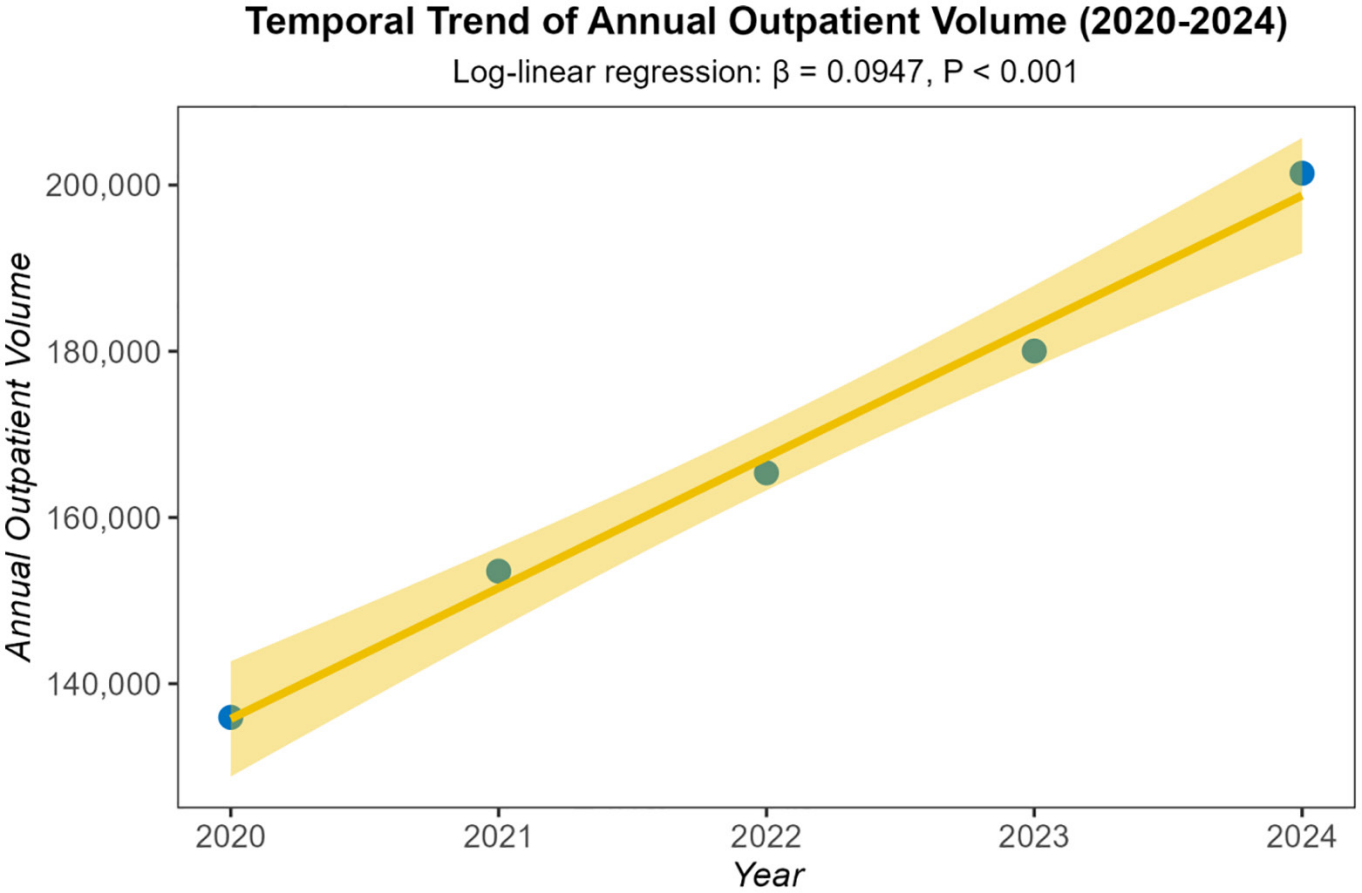
Log-linear regression analysis of annual outpatient volume in a tertiary TCM hospital (2020–2024). Log-linear regression analysis revealed a significant linear growth trend in annual outpatient volume (β = 0.0947, standard error = 0.0068, *t* = 13.93, *P* < .001), corresponding to an annual growth rate of 9.92%. The solid line represents the log-linear fitted curve, and the shaded area denotes the 95% confidence interval. TCM = Traditional Chinese Medicine.

Log-linear regression analysis confirmed a statistically significant upward linear trend in the annual outpatient volume after the exclusion of walk-in clinic and physical examination data (β = 0.0947, standard error = 0.0068, *t* = 13.93, *P* < .001), corresponding to an annual growth rate of 9.92% (95% CI: 8.14–11.86%; Fig. 3). The narrow 95% confidence interval indicated high stability and reliability of the estimated annual growth effect. Additionally, the significant positive regression coefficient is consistent with the continuous rising trend of annual outpatient visits observed in the descriptive statistical results.

### 3.3. Concentration degree analysis

**Figure 4.**
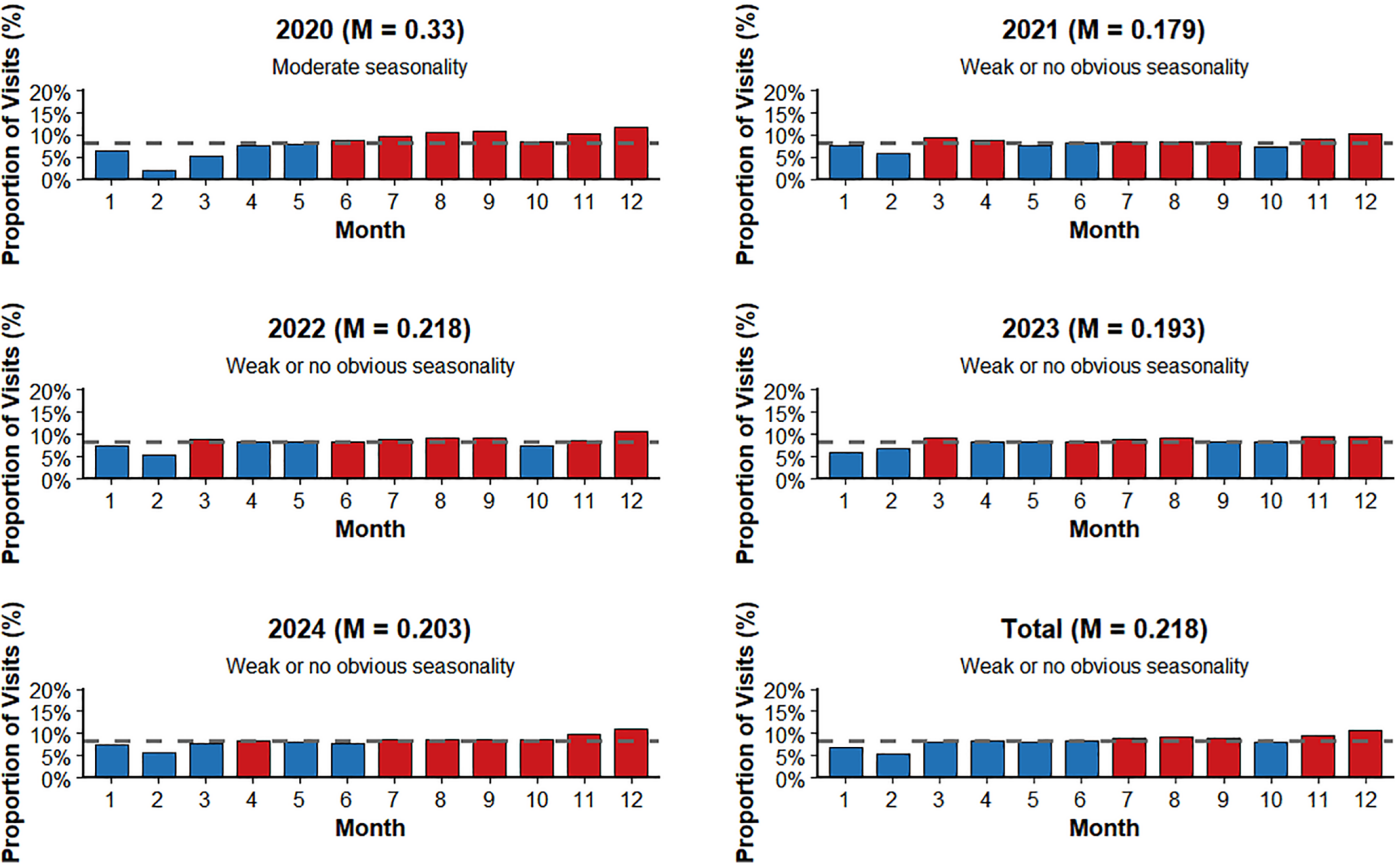
Monthly proportion distribution and seasonal concentration degree (*M* value) of outpatient volume in a tertiary TCM hospital (2020–2024). Monthly proportion of outpatient visits in each year; red bars indicate months with proportions above the annual average, and the gray dashed line represents the average outpatient visit proportion. TCM = Traditional Chinese Medicine.

The overall *M* value was 0.218, with *M* values ranging from 0.179 to 0.330 across the 5-year study period (Table 1 and Fig. 4). Monthly visit proportions above the annual average were concentrated in September–December, with the highest proportion observed in December (12.3%; Table 1). It should be noted that the highest monthly cumulative outpatient volume in December reflects the statistical result of monthly aggregate data, while the subsequent circular distribution analysis focuses on the temporal aggregation characteristics of daily outpatient volume throughout the whole year. The 2 analyses have different research perspectives: the former is the cumulative effect of outpatient volume in a single month, and the latter is the concentration distribution characteristics of daily outpatient flow, resulting in the identification of a “peak” between the 2 methods.

**Table 1:** Monthly distribution and seasonal concentration degree (*M* value) of outpatient volume in a tertiary TCM hospital (2020–2024).

Month	2020	2021	2022	2023	2024	Total
1	8863	11,642	12,013	10,487	14,942	57,947
2	2819	9140	8586	12,504	11,219	44,268
3	7186	14,561	14,435	16,408	15,615	68,205
4	10,261	13,718	13,560	14,826	16,970	69,335
5	10,665	11,801	13,765	14,952	16,401	67,584
6	12,110	12,778	13,880	15,163	15,385	69,316
7	13,372	13,198	14,511	15,788	17,213	74,082
8	14,239	12,983	15,398	16,361	17,285	76,266
9	14,983	12,941	15,254	14,648	17,442	75,268
10	11,616	11,036	12,083	14,716	17,108	66,559
11	13,959	14,136	14,331	17,111	19,563	79,100
12	15,879	15,605	17,560	17,073	22,287	88,404
*M*	0.330	0.179	0.218	0.193	0.203	0.218

The *M* value is a quantitative descriptive indicator for the intensity of seasonal aggregation of outpatient visits, with smaller values indicating weaker seasonal aggregation.

TCM = Traditional Chinese Medicine.

### 3.4. Circular distribution analysis

**Figure 5.**
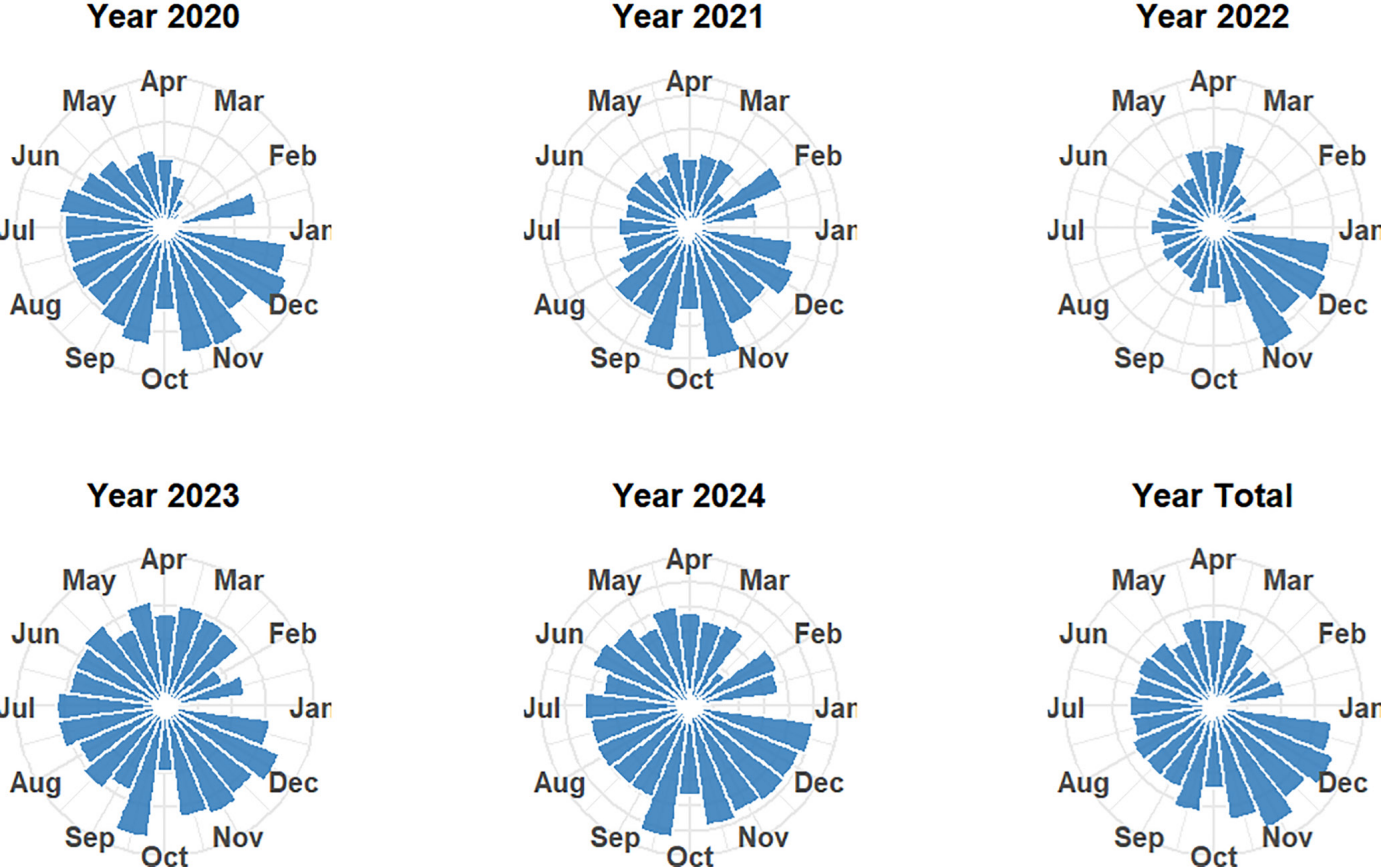
Circular rose plot of annual outpatient volume in a tertiary TCM hospital (2020–2024). Circular distribution of daily outpatient volume across 5 years, visualizing the temporal aggregation characteristics of outpatient visits. TCM = Traditional Chinese Medicine.

The overall mean angle was −74.8°, which corresponded to October 15, with the peak period spanning October 13 to 18 (Table 2). Annual peak days varied slightly across the study period: September 16 (2020), October 21 (2021), November 11 (2022), September 17 (2023), and September 30 (2024). Rayleigh test results (all *P* < .001) showed significant temporal aggregation; however, *r* values (0.034–0.184) indicated weak practical aggregation intensity (Table 2 and Fig. 5). Notably, the *Z* statistic (*Z* value of Rayleigh test [a sample-size dependent index for testing the significance of temporal aggregation]) of the Rayleigh test is sample-size dependent, and the extremely large *Z* values in this study stem from the massive daily observation data (1826 days). The *r* value, rather than the *Z* value, is the appropriate statistical index to characterize the actual intensity of temporal aggregation of outpatient visits.

**Table 2:** Peak day and period of outpatient volume via circular distribution (2020–2024).

Year	Mean angle (°)	Angular standard deviation (°)	Peak visit day	Peak visit period	*r* value	*Z* value	*P* value (Rayleigh)	*W* value	*P* value (Watson)
2020	−103.8	1.8	September 16	September 14–18	0.184	28,513.22	<.001	0.127	<.001
2021	−69.8	2.0	October 21	October 19–23	0.131	32,431.99	<.001	0.092	<.001
2022	−49.0	2.1	November 11	November 9–13	0.121	50,732.23	<.001	0.085	<.001
2023	−103.0	2.6	September 17	September 14–20	0.034	7391.90	<.001	0.024	<.001
2024	−89.7	2.3	September 30	September 28-Oct 3	0.069	16,790.99	<.001	0.048	<.001
Total	−74.8	2.2	October 15	October 13–18	0.098	1,25,726.84	<.001	0.069	<.001

## 4. Discussion

This 5-year retrospective study identified 2 key characteristics of outpatient visits in this tertiary TCM hospital: a steady annual growth of outpatient volume (annual growth rate = 9.92%) and seasonal temporal aggregation peaking in autumn. The overall *M* value was 0.218, and Rayleigh tests confirmed statistically significant temporal clustering (all *P* < .001). These findings mirror the growing public demand for TCM services and can support nursing management practices.

The annual increase in outpatient volume is consistent with national TCM development policies and people’s growing awareness of chronic disease management and health preservation,^[18]^ which puts forward higher requirements for outpatient nursing work. Inter-annual fluctuations of *M* values may be associated with changes in service demand and the policy environment, and this hypothesis requires further verification with disease composition data.

Notably, the concentration degree method applied in this study is a China-specific statistical tool for quantifying temporal event concentration (e.g., outpatient visits, disease onset).^[17]^ It differs fundamentally from the World Health Organization (WHO)-recommended Concentration Index, despite their similar nomenclature. The WHO-endorsed Concentration Index, typically paired with the Slope Index of Inequality, is a classic metric for measuring relative gradient health inequality across socioeconomic groups.^[19,20]^ For instance, Cao et al^[19]^ and Qu et al^[20]^ utilized the Slope Index of Inequality-Concentration Index indicator system to assess cross-country inequalities in autoimmune disease burden and regional disparities in cardiovascular disease burden among the elderly, respectively, with both studies focusing on socioeconomic-related distributive differences in health outcomes. In contrast, the concentration degree method focuses solely on the temporal aggregation of events, calculating a concentration index (*M* value) based on event frequency across equal time intervals (range: 0–1; closer to 1 indicates stronger temporal concentration) without involving any socioeconomic factors. This essential distinction underscores the critical need to clarify the definition and application context of similar-named statistical indicators in academic research to avoid misinterpretation.

The circular distribution method (also known as circular statistics) is widely applied to analyze periodic time-series data in medical research.^[17,21,22]^ The combined use of this method and the concentration degree method forms a comprehensive analytical framework for exploring the temporal distribution of medical service demand.

The autumn clustering of outpatient visits appears to align with the TCM core theory of “Tian Ren Xiang Ying (correspondence between heaven and humans).” The concept of “Qiu Zhuo Zao Qi (dryness dominates autumn)” is 1 possible explanation for the rise in autumn outpatient visits, as autumn dryness may induce respiratory diseases, xerosis syndrome, and skin disorders. Meanwhile, people’s pursuit of the TCM health preservation concept “Qiu Dong Yang Yin (nourishing yin in autumn and winter)” might also contribute to the increased use of TCM characteristic services.

The seasonal characteristics of TCM outpatient demand may reflect the differences in medical philosophies between TCM and Western medicine. TCM focuses on holistic conditioning and chronic disease management,^[23,24]^ and its service demand could be influenced by seasonal health preservation ideas and featured therapies.

### 4.1. Clinical implications

Based on the temporal distribution characteristics of TCM outpatient visits (steady growth and autumn-centered temporal aggregation), we put forward potential directions for outpatient nursing staffing arrangements. Nursing manpower can be properly adjusted during the autumn peak period to adapt to fluctuating outpatient demand. These ideas aim to integrate the TCM concept of “Zhi Wei Bing (preventive treatment of disease [a core TCM concept emphasizing disease prevention over post-illness treatment])” into daily nursing work and provide a reference for clinical nursing management.

### 4.2. Limitations

This study has several limitations. First, this is a single-center study conducted in 1 TCM hospital in Fujian Province, so the research findings have limited generalizability to other medical institutions and regions. We only adopted aggregated hospital-level data and did not obtain individual patient information or conduct an in-depth analysis on disease composition, which restricts further exploration of underlying causes and heterogeneity. Second, the trend analysis was based on only 5 annual observations, which restricted the statistical power of log-linear regression. External policies and post-pandemic changes in medical-seeking behavior may also serve as unmeasured confounding factors influencing outpatient volume. In addition, meteorological covariates were not incorporated, and potential changes in hospital capacity or staffing were not adjusted for as confounders. Third, the lack of stratification by TCM specialty or syndrome type limited the characterization of service-specific temporal patterns. Fourth, temporal autocorrelation in time-series data was not evaluated, which may have a certain impact on the interpretation of trend and clustering results.

### 4.3. Future directions

Future studies should adopt multicenter designs to compare outpatient temporal regularities across TCM hospitals of different regions and levels, enhancing the generalizability of findings. In-depth analysis of TCM-dominant diseases and characteristic therapies (e.g., cupping, moxibustion)^[24,25]^ is recommended, combined with TCM temporal medical theories (e.g., Twenty-Four Solar Terms)^[26]^ to develop more TCM-aligned demand prediction models. Additionally, exploring correlations between outpatient volume and meteorological factors or solar terms could provide deeper insights into the drivers of seasonal demand. Furthermore, future research can collect TCM outpatient service mix, disease composition, and other related data to verify the hypotheses about the causes of seasonal changes in outpatient volume and conduct empirical research on the effectiveness of the proposed nursing resource allocation optimization strategies.

In conclusion, this study clarifies the temporal distribution of outpatient visits in a TCM hospital, identifying steady growth and autumn-centered aggregation. Based on the findings, we put forward potential ideas for nursing staffing arrangements during peak periods. This study describes the temporal characteristics of outpatient visits in this TCM hospital and offers a reference for routine outpatient nursing management while embodying the TCM concept of preventive treatment of disease.

## Author contributions

**Data curation:** Qin Wang, Chunchun Chen, Huayu Fang, Jing Meng, Yan Cao.

**Formal analysis:** Qin Wang, Chunchun Chen.

**Funding acquisition:** Zhigang Lin.

**Methodology:** Qin Wang, Chunchun Chen, Xiaofang Wang, Zhigang Lin.

**Software:** Qin Wang, Chunchun Chen.

**Supervision:** Xiaofang Wang.

**Writing – original draft:** Qin Wang, Chunchun Chen, Huayu Fang, Jing Meng, Yan Cao.

**Writing – review & editing:** Xiaofang Wang, Zhigang Lin.
